# Water

**Published:** 1877-08-20

**Authors:** 


WATER.—Happily, the old custom of starving patients for water is giving away. Yet many old fogies, and nearly all the old women, still adhere to the custom. There may be exceptional cases, as in gastric inflammation, where water must be partially withheld, or given in small quantities with ice. Yet they are certainly rare, and it is a question whether or not a plenty of it taken cold and fresh from a well or spring would not give relief to the sufferer. Water is Nature's remedy for thirst, and there is no proper substitute for it. Recently we were called to a patient suffering from a remittent form of fever, with intense thirst, red and dry tongue, nausea and frequent vomiting, etc. For four days she had been thus suffering, no water being allowed, except a spoonful or two, which was vomited as soon as it became warm on the stomach. We ordered a dipper full of fresh, cold water to be given as often as she wanted it. The old nurse looked aghast at the idea, and the patient, though eager for the draft, trembled through fear of the consequences, having been taught to believe that it was dangerous by her old and favorite family physician, who had attended her once *for six weeks* in a similar attack, during the whole of which time she was not allowed a single full drink of water. The result in the above case was not only delightfully refreshing to the patient, but soon brought about a favorable change in her condition. The heat and thirst were diminished, the secretions and emunctories went to work, remedies which before were rejected or inefficient were absorbed and appropriated, and the patient was soon restored to health.

